# Complicated Extra-Abdominal Desmoid Tumor Associated With Pregnancy in a Patient With a History of Bilateral Mastectomy: A Case Report

**DOI:** 10.7759/cureus.88308

**Published:** 2025-07-19

**Authors:** Franscisco Ibargüengoitia-Ochoa, Cintia M Sepúlveda-Rivera, Silvia F Estrada-Rivera, Verónica Villavicencio-Valencia, Ana Paulina Melendez-Fernandez, Dorian Y Garcia-Ortega, Juan I Sánchez-Charvet

**Affiliations:** 1 Obstetrics Department, National Institute of Perinatology, Mexico City, MEX; 2 Gynecology Department, National Institute of Perinatology, Mexico City, MEX; 3 Surgical Oncology Department, National Institute of Cancerology, Mexico City, MEX

**Keywords:** abdominal wall tumor, bilateral mastectomy, desmoid tumor, phyllodes tumor, pregnancy

## Abstract

Desmoid-type fibromatosis is an uncommon, locally aggressive, non-metastatic soft-tissue neoplasm that accounts for <0.03% of all tumors; breast or multifocal disease is particularly rare, and the hormonal milieu of pregnancy may accelerate tumor growth. We report an 18-year-old primigravida who underwent bilateral mastectomy at age 17 for presumed phyllodes tumors that were subsequently reclassified as bilateral breast desmoid tumors on histopathologic review. During her current pregnancy, she developed two rapidly enlarging abdominal-wall masses, one becoming necrotic and infected, necessitating multidisciplinary evaluation. At 33 weeks’ gestation, a cesarean section was performed via a left paramedian incision that avoided the tumors, delivering a viable male infant; the mother was then referred to a tertiary oncologic center for definitive management. This case illustrates the diagnostic uncertainty, potential for rapid progression, and need for individualized, multidisciplinary care when desmoid tumors arise or recur during pregnancy to balance maternal oncologic control with fetal well-being.

## Introduction

Desmoid-type fibromatosis (DTF) is a rare, non-metastatic yet locally aggressive soft-tissue neoplasm that arises from musculo-aponeurotic structures. It accounts for ≈0.03% of all neoplasms and 3% of soft-tissue tumors [1,2]. DTF lesions infiltrate along fascial planes, recur in up to 30% to 70% of cases despite macroscopically complete excision, and are classified anatomically as intra-abdominal, abdominal-wall, or extra-abdominal [3,4].

Although most DTFs are sporadic and harbor activating CTNNB1 mutations, pregnancy-associated tumors represent a distinct clinical subset: estrogen-receptor-β overexpression can amplify Wnt/β-catenin signaling, while mechanical stretching of the gravid abdominal wall may further stimulate myofibroblast proliferation [5]. Reported prevalence is low (≈0.2 - 0.3% of all pregnancies), yet rapid third-trimester enlargement occasionally necessitates preterm delivery.

Desmoid tumors of the breast are particularly uncommon, comprising ≈0.2% of breast neoplasms [6,7]. Bilateral or multicentric presentations are exceptionally rare, and misdiagnosis as phyllodes tumors is well documented owing to overlapping spindle-cell histology.

## Case presentation

**Figure 1:**
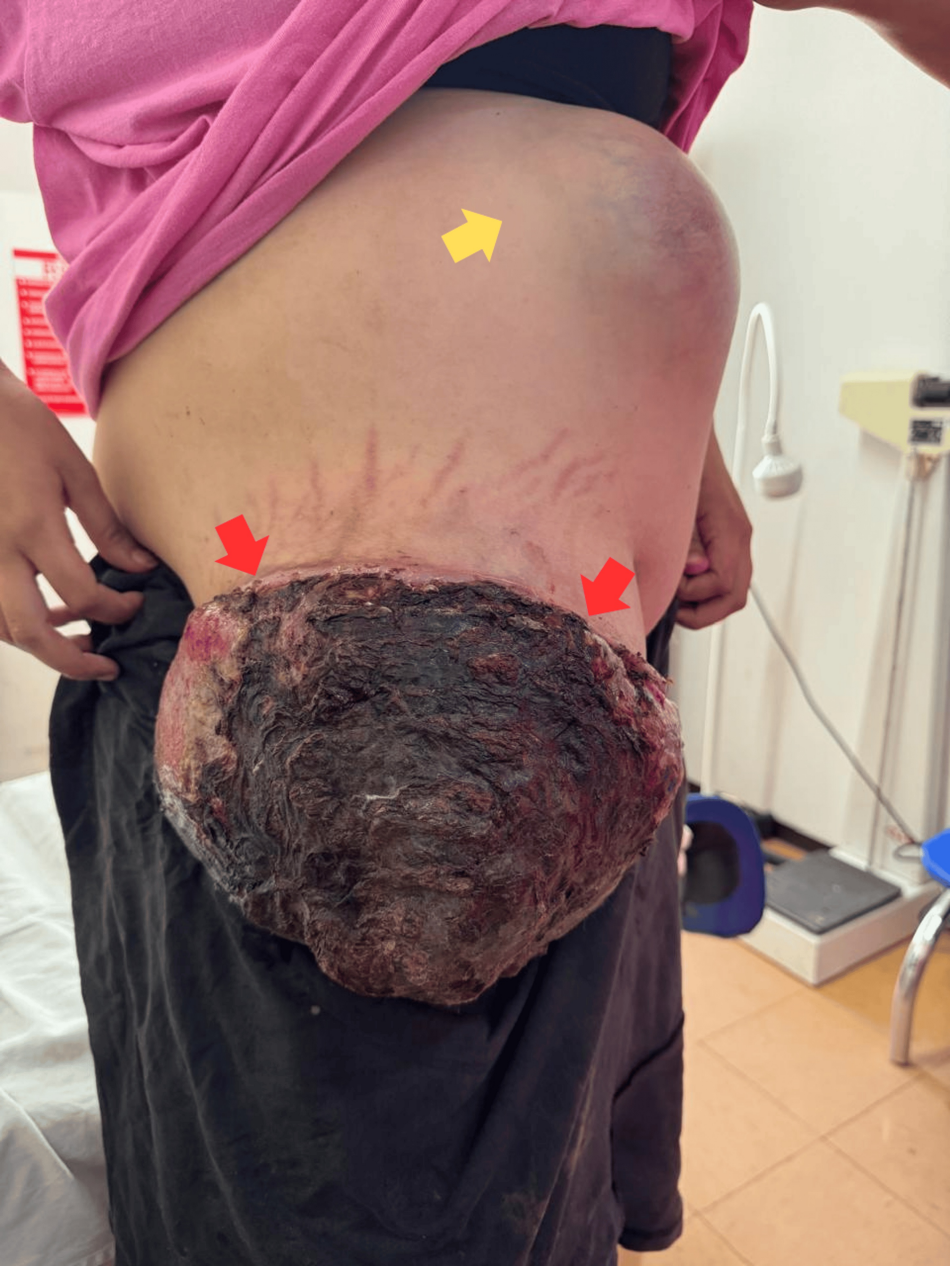
Clinical photograph for case documentation. Red arrows indicate a large exophytic tumor on the right side, with areas of necrosis and hemorrhage. The yellow arrow indicates a second tumor on the left side.

An 18-year-old primigravida, who had undergone bilateral mastectomy at age 17 for suspected phyllodes tumors, was referred at 32 + 6 weeks’ gestation with a rapidly enlarging abdominal-wall mass clinically suspected to be a liposarcoma. Physical examination revealed a firm, non-tender, mobile tumor in the right abdominal wall measuring approximately 23 cm in diameter and a second lesion on the left measuring 13 cm. Written informed consent for publication of all clinical photographs, case details, and future germline genetic testing was obtained from the patient and her legal guardian. In line with Desmoid Tumor Working Group and European Society for Medical Oncology recommendations, a planned caesarean delivery was performed at 33 + 3 weeks to balance maternal morbidity risk with fetal maturity (Figure 1).

A computed tomography (CT) scan was obtained at 32 + 3 weeks’ gestation using a low-dose, single-phase protocol in accordance with American College of Radiology-Society for Pediatric Radiology guidelines for imaging pregnant patients. Automatic exposure control with tube-current modulation (120 kVp, 80 mAs) and a lead apron over the maternal pelvis limited the estimated fetal effective dose to 4.8 mGy, well below the 50 mGy threshold associated with deterministic effects and consistent with as low as reasonably achievable (ALARA) principles. The CT demonstrated two well-defined cystic lesions within the abdominal-wall adipose tissue: one in the left upper quadrant measuring 13 × 10 cm and another in the right lower quadrant measuring 23 × 20 cm. Upon admission, the lower mass measured 30 × 25 cm and showed exophytic growth, surface necrosis, hemorrhagic areas, and purulent discharge. Culture was positive for *Escherichia coli*. Obstetric ultrasound confirmed a live singleton fetus weighing approximately 1700 g, with normal amniotic fluid and a posterior placenta.

**Figure 2:**
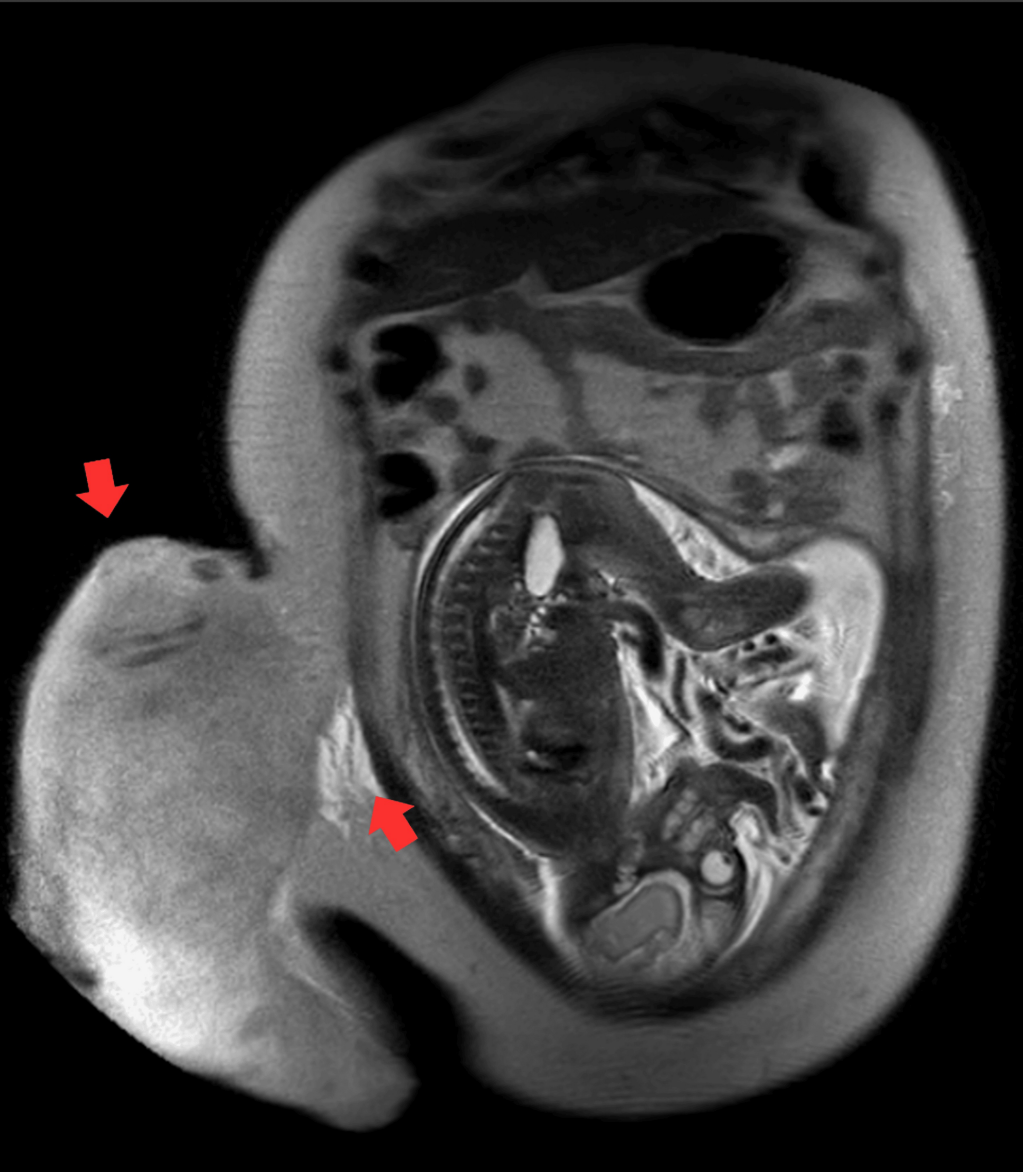
Sagittal MRI view shows a large muscle-derived tumor measuring 25 × 18 × 21 cm. MRI: Magnetic resonance imaging

Magnetic resonance imaging (MRI) revealed a 25 × 18 × 21 cm subcutaneous lesion, likely with myxoid content, without invasion of adjacent structures. Histopathologic review of previous breast surgery slides and a biopsy of the upper abdominal mass both confirmed desmoid-type fibromatosis (Figure 2).

**Figure 3:**
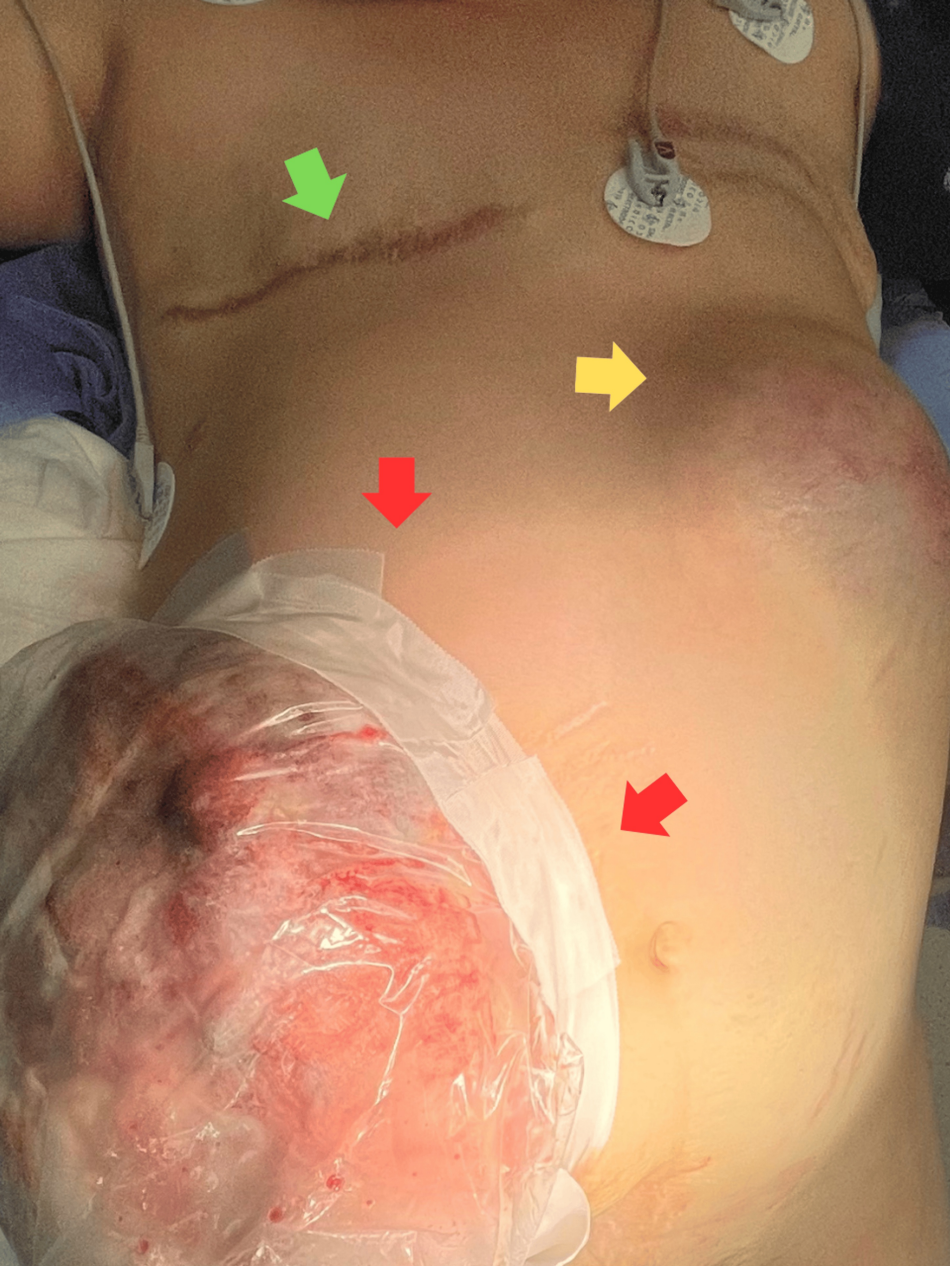
Pre-cesarean photograph. The green arrow marks the mastectomy scar. Yellow arrow: left-sided tumor. Red arrows: isolated right-sided tumor prior to surgical asepsis.

Following multidisciplinary evaluation by obstetrics, neonatology, and oncology teams, pregnancy termination was recommended. A preoperative clinical photograph was obtained for documentation, illustrating the surgical field prior to aseptic preparation (Figure 3). At 33 weeks and three days of gestation, a cesarean section was performed under regional anesthesia through a left paramedian incision to avoid the affected area. Intraoperatively, the lesion was confined to the abdominal wall without evidence of intraperitoneal extension.

**Figure 4:**
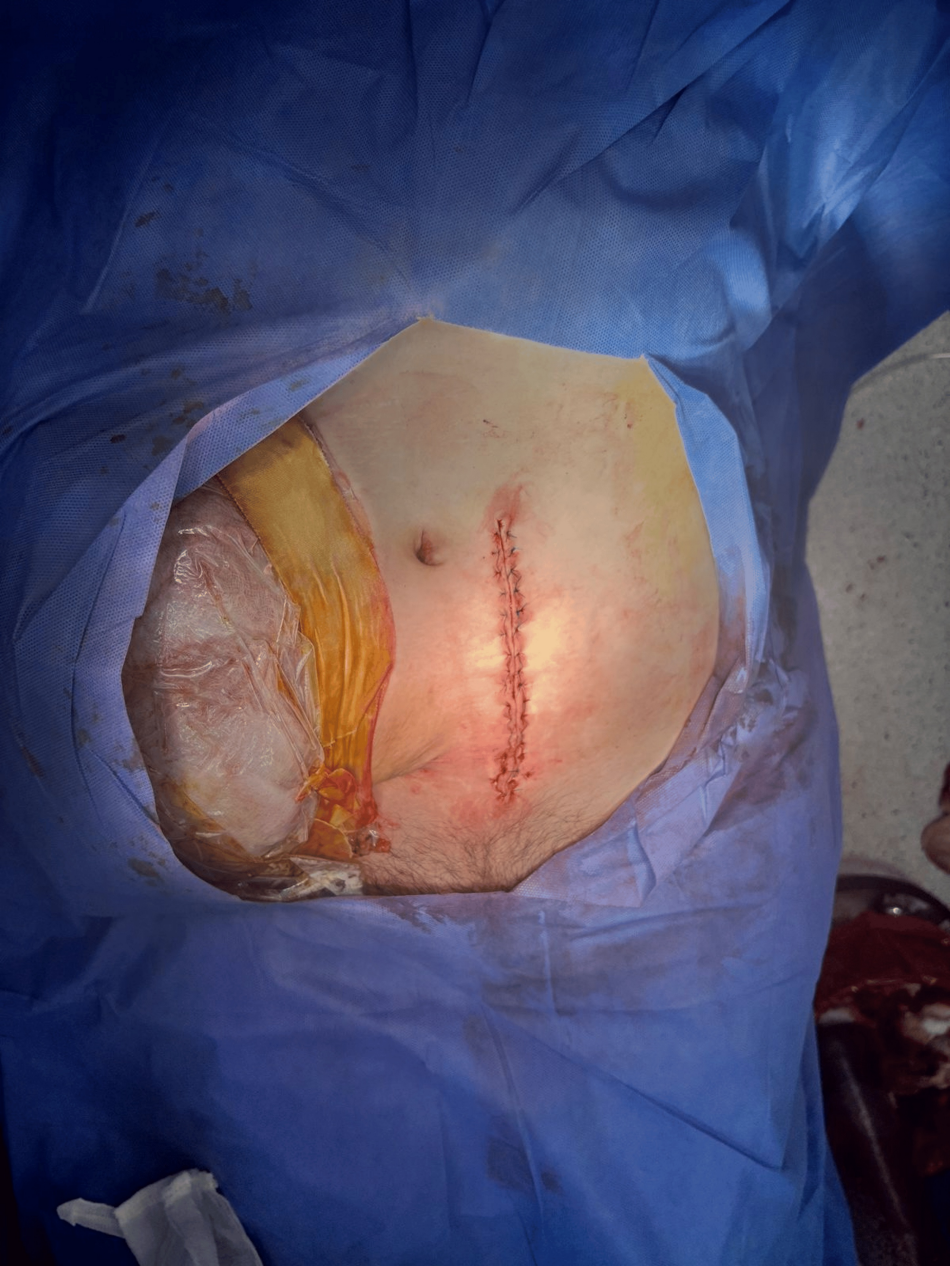
Post-cesarean photograph. Left paramedian incision visible. Desmoid tumors are isolated from surgical site.

A male neonate was delivered, weighing 1750 g, with Apgar scores of 4 and 9 at 1 and 5 minutes, respectively. Gestational age, as assessed by the Capurro method, was estimated at 33.3 weeks. The newborn was admitted to the intermediate care unit with a diagnosis of a late preterm infant, appropriate for gestational age, but classified as having low birth weight. He subsequently developed grade 2 respiratory distress syndrome and received a single dose of exogenous surfactant, with a favorable response. A post-cesarean clinical photograph was obtained, showing the left paramedian surgical incision and the desmoid tumor clearly separated from the operative site (Figure 4).

**Figure 5:**
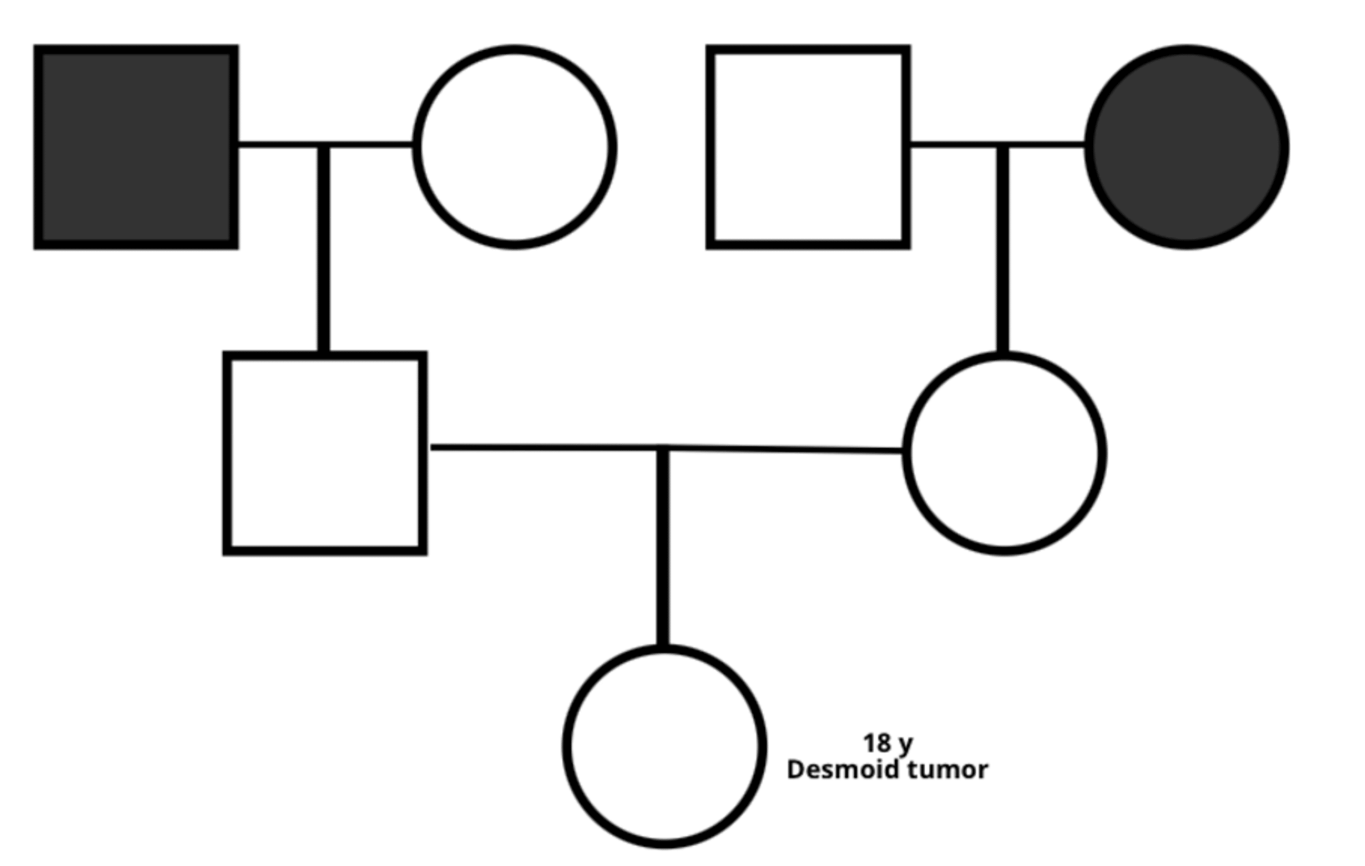
Three-generation pedigree showing an 18-year-old female with a desmoid tumor and no other affected relatives. The image is created by the author.

Because the patient had bilateral breast desmoid-type tumors resected at age 17, rapidly progressive multifocal abdominal-wall lesions in the current pregnancy, and a young age (18 years), she met two Chompret criteria and was referred for clinical-genetics evaluation to exclude Li-Fraumeni syndrome. The archived mastectomy specimens were re-examined: H&E sections showed uniform spindle-cell fascicles with infiltrative borders, and immunohistochemistry demonstrated diffuse nuclear β-catenin and focal smooth-muscle-actin positivity with negative cytokeratin, S-100, desmin, and CD34. Targeted sequencing of CTNNB1 exon 3 confirmed a pathogenic p.S45F missense mutation, while no argon plasma coagulation (APC) alteration was detected. These histopathologic and molecular findings satisfy current WHO and Desmoid Tumor Working Group diagnostic criteria, supporting reclassification of the breast lesions as desmoid-type fibromatosis. A three-generation pedigree is shown in Figure 5, revealing no personal or family history of cancer or known hereditary syndromes.

In the early postoperative period, the patient was referred to a tertiary center. A review of a core biopsy from the right lower quadrant mass confirmed desmoid-type fibromatosis, showing diffuse nuclear β-catenin, “double-rail” smooth-muscle-actin and vimentin positivity, focal factor XIIIa staining, Ki-67 < 1%, and a pathogenic CTNNB1 p.T41A mutation, with desmin, S-100, cytokeratin AE1/AE3, and CD34 all negative. After multidisciplinary discussion, first-line systemic therapy with the PD-1 inhibitor pembrolizumab (200 mg IV every three weeks, started four weeks postpartum under an expanded-access protocol) was selected on the basis of emerging response rates in progressive desmoid-type fibromatosis and its favorable safety profile for lactating women. Three-month follow-up imaging demonstrated radiological disease stability, the patient remained asymptomatic (ECOG 0) and free of immune-related adverse events, and surgical re-evaluation is planned at six months.

## Discussion

Desmoid tumors are locally aggressive myofibroblastic neoplasms arising from deep mesenchymal tissues. Despite their benign histology and lack of metastatic potential, their infiltrative behavior, high recurrence rate, and capacity to compromise vital structures make them clinically significant, particularly in young patients and women of reproductive age [1,2].

Various factors have been implicated in their pathogenesis, including hormonal changes, trauma, prior surgery, and pregnancy. The link to pregnancy is well documented and thought to be related to elevated estrogen levels and mechanical strain on the abdominal wall [3,4]. Clinical behavior during pregnancy is variable: some tumors remain stable or regress spontaneously, while others may grow rapidly or develop complications such as necrosis, hemorrhage, or superinfection, as seen in our patient [5,6].

In this case, an 18-year-old primigravida developed two abdominal-wall masses that enlarged markedly during late pregnancy. Low-dose thoraco-abdominal CT at 32 + 3 weeks’ gestation demonstrated a right-lower-quadrant lesion measuring 23.4 × 20.4 cm and a left-upper-quadrant lesion measuring 13.1 × 10.7 cm. A pelvic MRI performed just five days later (33 + 1 weeks) documented interval growth of the right-sided mass to 25.4 × 18.4 × 21.3 cm, an approximate 30% increase in maximal diameter, and of the left-sided mass to 12.6 × 14.3 × 8.8 cm. Because the larger tumor showed central necrosis with purulent discharge, a planned cesarean delivery was undertaken at 33 + 3 weeks. Intraoperatively, both lesions were confined to the superficial fascia without intraperitoneal infiltration, and postpartum clinical measurements confirmed stable dimensions.

A multicenter retrospective study involving 62 patients with pregnancy-related desmoid fibromatosis reported that 16% were diagnosed during pregnancy. The most common location was the abdominal wall (74%), with initial management being active surveillance in 61% of cases. The progression rate was 33%, and the postoperative recurrence rate remained low (17%), even with positive margins [7,8]. Another large series of pregnancy-related desmoid tumors found that obstetric outcomes were comparable to the general population, supporting pregnancy continuation in patients with stable or previously treated disease under specialized monitoring [9-11].

Neonatal outcomes are generally favorable in reported cases; nevertheless, when preterm delivery is necessary because of aggressive tumor growth, complications such as respiratory distress syndrome and low birth weight can occur, as in our neonate (birth weight 1,750 g at 33 + 3 weeks’ gestation, Apgar scores 4 and 9 at 1 and 5 minutes, respectively). The infant required a single dose of surfactant for grade 2 respiratory distress and progressed uneventfully thereafter.

As part of the work-up, the patient was referred for clinical-genetics evaluation because of her age (18 years), prior bilateral breast desmoid-type fibromatosis resected at 17 years, and new multifocal abdominal-wall lesions met two Chompret criteria for Li-Fraumeni syndrome. Desmoid-type fibromatosis (DTF) arises in three principal molecular settings that shape multifocality and recurrence: (i) sporadic CTNNB1-mutant tumors (~85% of DTF), in which the S45F hotspot variant carries ≈70% 5-year local-recurrence risk, whereas the T41A mutation identified in our patient confers a lower, yet still significant, risk of ≈25%, amplified by estrogen exposure and prior surgery; (ii) APC germ-line mutations in familial adenomatous polyposis/Gardner syndrome (10-15%), where up to 30% of carriers develop often multifocal, intra-abdominal DTF with ≈50% recurrence irrespective of margins; and (iii) the rarer TP53 germ-line mutations of Li-Fraumeni syndrome, in which impaired p53-mediated growth arrest may fuel rapid growth and early relapse, although DTF penetrance is not well defined. After bilateral breast DTF resection, pooled data indicate a weighted five-year recurrence rate of ≈28% (range 12-45%), modulated by mutation subtype, positive margins, and high-estrogen states (pregnancy, combined oral contraceptives). These considerations informed our strategy of close imaging surveillance and postpartum systemic therapy rather than immediate re-excision [12,13].

Regarding systemic therapy, the patient was considered a candidate for pazopanib, a multikinase inhibitor targeting VEGFR, PDGFR, and c-KIT. In a multicenter study, pazopanib showed a disease control rate of 95.1%, median tumor shrinkage of 8.5%, and one- and two-year progression-free survival rates of 82% and 80%, respectively. Toxicity was manageable, with hypertension and diarrhea being the most frequent adverse events [14-16].

Tyrosine kinase inhibitors have now replaced cytotoxic agents as first-line treatment for progressive disease. Sorafenib and pazopanib are validated options, with the latter demonstrating effectiveness even outside clinical trials according to recent observational studies [17-20].

## Conclusions

Desmoid-type fibromatosis (DTF) arising in pregnancy, although uncommon, can enlarge quickly and mimic sarcoma, making early tissue confirmation with β-catenin immunostaining and CTNNB1 sequencing, quantitative serial imaging, and tight multidisciplinary collaboration indispensable. In our CTNNB1 p.T41A-mutant case, bilateral breast DTF recurred in the abdominal wall during the third trimester; prompt histologic diagnosis and growth-rate documentation guided a planned caesarean at 33 + 3 weeks that protected both mother and fetus, while postpartum pembrolizumab kept the disease stable without immune-related toxicity. This experience underscores two practical insights echoed in emerging literature: pregnancy-associated DTF remains non-metastatic despite aggressive local behavior, and immune-checkpoint blockade offers a feasible postpartum alternative when surgery would entail excessive morbidity. Accumulating larger case series will be vital to fine-tune obstetric timing, optimize systemic-therapy sequencing, and ensure long-term maternal and neonatal safety.
